# Correction: From theory to practice: An integrated TTF-UTAUT study on electric vehicle adoption behavior

**DOI:** 10.1371/journal.pone.0306761

**Published:** 2024-07-03

**Authors:** Ayed Alwadain, Suliman Mohamed Fati, Kashif Ali, Rao Faizan Ali

There are errors in the Funding statement. The correct Funding statement is as follows: Research is supported by Researchers Supporting Project number (RSP2024R309), King Saud University, Riyadh, Saudi Arabia. The authors would also like to thank Prince Sultan University, Saudi Arabia for their support in data collection.
