# Supplementary material for: From theory to practice: An integrated TTF-UTAUT study on electric vehicle adoption behavior
Source: PLoS One. 2024 Mar 12;19(3):e0297890. doi: 10.1371/journal.pone.0297890 (PMC10931523; doi:10.1371/journal.pone.0297890)
Supplement: S1 File — This file highlights the past literature studies on TTF and UTAUT relationship. (DOCX) [file pone.0297890.s001.docx]

| **Sr#** | **Authors** | **Evidence type** | **Purpose** | **Theory** | **Industry type** | **Sampling** | **Unit of analysis** | **Country** | **Findings** |
| --- | --- | --- | --- | --- | --- | --- | --- | --- | --- |
| 1 | Liu et al. (2023) | Empirical | To study the patient attitudes towards the usage and adoption of accessible electronic health records. | TTF, UTAUT, and Delone & McLean IS success model | Healthcare | Random | Patients | China | The results indicated that performance expectance, effort expectance, social influence and TTF are essential predictors of behavioural intention. |
| 2 | Shirolkar and Kadam (2023) | Empirical | To study the adoption and utilization of online examination portal in Indian universities. | UTAUT and TTF | Education | Convenience | Student | India | The outcomes indicated that UTAUT factors have direct effect on student adoption and TTF has a moderating impact on the relationships. |
| 3 | Sediyaningsih et al. 2023 | Empirical | To study the usage intention of metaverse technology in digital library services. | UTAUT, Unified information theory, and TTF | Higher education |  | Staff & students | Indonesia | The results suggested that TTF and UTAUT and ISS theories have well-explained the intention to use metaverse in higher education. |
| 4 | Uymaz et al. (2023) | Empirical | To study the BI to use AI doctor at the individual level at primary, secondary and teritary care levels. | UTAUT and TTF | Healthcare |  | Individual staff | Turkey | The outcome depicted that TTF has significant linkages with UTAUT variables and BI. |
| 5 | Dayour et al. (2023) | Qualitative (Interview) | To study the impact of ICT services on SME tourism and hospitality enterprises in Ghana | UTAUT2, TTF, Diffusion of innovation | Tourism & hospitality | Purposive | Hotel owner/manager | Ghana | The findings depicted that adoption of IT leads to the sustainability and survival of SMEs in tourism and hospitality industry. |
| 6 | Tian and Yang (2023) | Empirical | To examine the student interpreters BI to use interpreting technology in China. | UTAUT and TTF | Education | Purposive | Student | China | The result indicated that TTF and UTAUT (performance expectancy) have the most significant relationship. |
| 7 | Cai et al. (2023) | Empirical | To study the process of high prefabrication level technologies adoption form the enterprise perspective in China | UTAUT and TTF | Construction |  | Professional | China | The constructs of TTF have significant association with UTAUT variables. |
| 8 | Hajj et al. (2023) | Empirical | To study the role of TTF, sense of virtual community and CI to use the E-government in Lebanon. | TTF, UTAUT and Sense of virtual community | Government | SRS | Public/citizen | Lebonen | The study indicated that UTAUT has a mediating role between TTF and sense of virtual community toward the adoption of e-government in Lebonen. |
| 9 | Khashan et al. (2023) | Empirical | To study the retailing customers adoption of augmented reality apps in Egypt. | UTAUT2 and TTF | Retailing | Snowball | Customer | Egypt | The outcomes depicted that TTF and UTAUT2 positively impact the customer BI to adopt AR apps in Egypt. |
| 10 | Alkhwaldi et al. (2022) | Empirical | To analyze the adoption of HR information systems in Jordan public sector. | UTAUT and TTF | Public sector | Convenience | HR managers | Jordan | The findings highlight that UTAUT variables have a direct impact on user intention to adopt HRIS in the public sector and TTF has a mediating role between UTAUT and adoption intention. |
| 11 | Sun and Guo (2022) | Empirical | To study the adoption of digital museum usage behavior in China. | UTAUT2 and TTF | Tourism | None | Tourist | China | Both UTAUT-TTF can server an essential role in predicting consumer digital museum use behaviour. |
| 12 | Kim et al. (2022) | Empirical | To investigate the drivers of buy online & pick-up store (BOPS) usage intention of omnichannel consumers in the automotive retail sector. | UTAUT and TTF | Automotive | None | Customers | South Korea | Both UTAUT-TTF can server an essential role in predicting consumer behaviour. |
| 13 | Al-Rahmi et al. (2022) | Empirical | To evaluate the role of social media for teaching & learning in higher education. | UTAUT and TTF | Education | Stratified random | University student | Malaysia | The integration of information system theories (UTAUT-TTF) promote students active learning and enable them to exchange information and knowledge more efficiently. |
| 14 | Wang et al. (2022) | Empirical | To study the students intention to use tablet computers in China | UTAUT and TTF | Education | Convenience | Student | China | The factors like hedonic motivation and TTF are predictors among village students and habit and TTF are the most significant factors in urban areas students. |
| 15 | Abdekhoda et al. (2022) | Empirical | To determine the factors in adoption of e-learning in healthcare. | UTAUT and TTF | Healthcare | Random | University faculty | Iran | The integration of UTAUT and TTF explicitly explain the faculty intention to adopt e-learning. |
| 16 | Kang et al. (2022) | Empirical | To examine the behavioural intention to adopt smart home healthcare services in South Korea. | UTAUT and TTF | Healthcare | Random | Customers | South Korea | The findings indicated that UTAUT and TTF help to understand the user intention to adopt smart healthcare services. |
| 17 | Gu et al. (2021) | Empirical | To examine the adoption of e-health technology in Pakistan. | UTAUT and TTF | Healthcare | None | Patients | Pakistan | The results indicated that adoption of e-health technology is mostly influenced by the factors of UTAUT and TTF. |
| 18 | Sani et al. (2021) | Empirical | To study the factors affecting the adoption of big data analytics. | UTAUT, TTF and Initial trust model | Public sector | Random | Employees | Malaysia | The empirical findings indicated that both UTAUT and TTF are valid frameworks to adopt big data analytics in Malaysian government agencies. |
| 19 | Shahbaz et al. (2021) | Empirical | To examine the adoption of big data analytics-environmental air pollution management systems. | UTAUT and TTF | Public sector | Random | Employees | Pakistan | Both UTAUT and TTF are the stronger predictors of BI than either UTAUT or TTF alone. |
| 20 | Wan et al. (2020) | Empirical | To study the student intention of use massive open online courses. | UTAUT and TTF | Education | Random | Student | China | The results indicated that the factors of UTAUT model have direct impact on student open course learning, and TTF has indirect effect on continued intention. |
| 21 | Zhao and Bacao (2020) | Empirical | To determine the adoption factors related to food delivery apps. | UTAUT, TTF, and Expectancy confirmation model | Food | None | Consumer | China | The findings show that both frameworks (UTAUT-TTF) have direct and indirect positive impacts on user intention for food delivery mobile application. |
| 22 | Wang et al. (2020) | Empirical | To examine and understand the consumer acceptance of healthcare wearable devices. | UTAUT and TTF | Healthcare | Convenience | Consumer | China | The empirical results indicated that UTAUT factors (PE. EE, FC & SI) and TTF positively impacts BI and together accounted 68% of its variance. |
| 23 | Alazab et al. (2020) | Empirical | To investigate the adoption of blockchain in supply chain management. | UTAUT-TTF, Information system success (ISS) model | Manufacturing | None | SC Managers | Australia | The results indicated that IS theories (UTAUT-TTF-ISS) positively affect the supply chain managers to adopt blockchain. |
| 24 | Sahid et al. (2020) | Empirical | To examine the factors affecting the IT professional behavioural intentions in adopting big data analytics in Malaysian public sectors. | UTAUT-TTF and Initial trust model | Public sector | Random | Employees | Malaysia | Both UTAUT and TTF are the stronger predictors of BI than either UTAUT or TTF alone. |
| 25 | Hilal et al. (2019) | Conceptual | To develop a hybrid model for building information modelling adoption in facilities management. | UTAUT and TTF | Construction | None | None | None | The outcomes indicated that the acceptance and adoption of building information modelling adoption in facilities management is improved by UTAUT and TTF. |
| 26 | Isaac et al. (2019) | Empirical | To examine the adoption of internet usage among Yemen government organizations. | UTAUT and TTF | Public sector | None | Employees | Yemen | The integration of UTAUT and TTF explicitly explains the government intention to adopt the internet, and both frameworks explain 29% of variance. |
| 27 | Paulo et al. (2017) | Empirical | To examine the adoption of mobile augmented reality in tourism. | UTAUT2 and TTF | Services | None | Customers | Portugal | The empirical findings highlight that both frameworks explain 72% variance to adopt augmented reality and 45% user behaviour. |
| 28 | Tarhini et al. (2016) | Empirical | To analyze the factors that facilitate or hinder the usage of internet banking in Lebanon. | UTAUT and TTF | Banking | Convenience | Customers | Lebonan | The findings highlight that performance expectancy, social influence, perceived credibility and TTF are the essential predictors to explain customer behavioural intention to use Lebanon banking services. |
| 29 | Afshand and Sharif (2016) | Empirical | To examine the mobile banking adoption among university students in Pakistan. | UTAUT-TTF and Initial trust model | Education | Convenience | University student | Pakistan | The results indicated that TTF, UTAUT and initial trust model (ITM) are important framework to explain the mobile banking adoption. |
| 30 | Park et al. (2015) | Empirical | To examine the factors that affect the consumers intentions to use a revolutionary technology-driven product. | UTAUT and TTF | Online retail | Random | Customers | USA | Both frameworks (UTAUT and TTF) positively explain the consumers intention to adopt technology-driven products. |
| 31 | Oliveira et al. (2014) | Empirical | To exaime the adoption of mobile banking in Portugal. | UTAUT-TTF and Initial trust model | Banking | None | Customers | Portugal | The results indicated that TTF, UTAUT and initial trust model (ITM) are important framework to explain the mobile banking adoption. |
| 32 | Chang (2013) | Empirical | To explain the student behavioral intention of using library mobile applications. | UTAUT and TTF | Education | Convenience | University student | Taiwan | The findings indicated that UTAUT has a direct affect on student behaviour, and TTF has a moderating role on the relationships. |
| 33 | Pai and Tu (2011) | Empirical | To investigate the acceptance and usage of customer relationship management systems in Taiwan service industry. | UTAUT and TTF | Services | None | Employees | Taiwan | Both theoretical frameworks are important to adopt new and innovative technologies. |
| 34 | Zhou et al. (2010) | Empirical | To examine the mobile banking adoption among Chinese customers. | UTAUT and TTF | Banking | Random | Customers | China | The outcomes highlight that both UTAUT and TTF are essential theoretical framework to explain the mobile banking user adoption. |
