# Supplementary material for: From theory to practice: An integrated TTF-UTAUT study on electric vehicle adoption behavior
Source: PLoS One. 2024 Mar 12;19(3):e0297890. doi: 10.1371/journal.pone.0297890 (PMC10931523; doi:10.1371/journal.pone.0297890)
Supplement: Attachment — Submitted filename: Review.docx [file pone.0297890.s002.docx]

The manuscript’s aims are sound, and the structure of the study, somewhat, is appropriate. Also, the findings of the study are considerable.

Nevertheless, I suggest the manuscript could be improved significantly by some revisions that are outlined below. I suggest the manuscript’s publications in your journal after the editors are satisfied the authors have made the suggested revisions. The suggested revisions (detailed below) include:

- In Introduction section, I suggest the authors clearly addressed that why he/she carried out this study? The gap should be addressed in introduction. However, the authors refer to the gap, but more information is needed.
- In the literature section, there are many study which addressed this issue. I suggest the authors mentioned them in literature review section.
- I suggest the manuscript could be improved significantly by more description of findings and cite to more document in each specific section.
- The contribution of knowledge is not clear, very well. The author should identify the lessons that we can learn from his/her study.
- The originally / novelty of the study is not clear very well.
