# Supplementary material for: From theory to practice: An integrated TTF-UTAUT study on electric vehicle adoption behavior
Source: PLoS One. 2024 Mar 12;19(3):e0297890. doi: 10.1371/journal.pone.0297890 (PMC10931523; doi:10.1371/journal.pone.0297890)
Supplement: Attachment — Submitted filename: Response to Reviewers-R1.docx [file pone.0297890.s003.docx]

**Original Manuscript ID:** **PONE-D-23-27266R1**

**Original Article Title: “From Theory to Practice: An integrated TTF-UTAUT study on Electric Vehicle Adoption Behavior**”

**To:** Plos One Editor

**Re:** Response to reviewers

Dear Editor,

Thank you for allowing a resubmission of our manuscript, with an opportunity to address the reviewers’ comments.

We are uploading (a) our point-by-point response to the comments (below) (response to reviewers), (b) an updated manuscript with yellow highlighting indicating changes, and (c) a clean updated manuscript without highlights (PDF main document).

Best regards,

All study authors

**Reviewer#1, Comment # 1:** **Dear author main theories that you have used in designing research framework are not technically linked and therefore unable to recommend your work, For instant TTF theory is not properly linked similarly, what you mean effort expectancy where ? in driving ? or adoption ?**

**Author response: Thanks for your concern.**

**Author action:** We appreciate your thoughtful review and constructive comments on the research presented. Your feedback has been instrumental in refining the theoretical foundations of the study. I would like to provide clarifications and address the concerns you raised regarding technical linkages of main theories, particularly TTF.

- Integration of TTF with UTAUT theory

“*This research integrates the UTAUT and TTF to explain the user adoption of EVs from both perspective including TTF and technology perceptions. According to Zhou et al. (2010) argued that individual variances of UTAUT (45.7%) and TTF (43.3%) lower than the integrated model of UTAUT-TTF (57.5%). This highlight the advantage of integrated model over individual models. Khashan et al. (2023) argued that the integration of both theories is crucial, emphasizing that user preference to adopt new technologies goes beyond mere perceptions. It also hinges on how effectively the characteristics of those technologies align with the tasks they are intended to assist users in completing. Several studies have found a strong association among TTF and UTAUT constructs, which may facilitate adoption in automotive (Kim et al., 2022), banking (Tarhini et al., 2016), construction (Cai et al., 2023), education (Tian and Yang, 2023), healthcare (Uymaz et al., 2023), and public sector (Alkhwaldi et al., 2022) as shown in Table 1. Nevertheless, there is a dearth of empirical evidence supporting the application of the integrated framework in the EVs domain. This points to a theoretical gap in previous research, which can be addressed by incorporating it into the current study to fill this void*.” Furthermore, a the below table explain the integration of TTF and UTAUT in the prior literature.

| **Theoretical framework** | **Industry/sector type** | **Country** | **Sampling technique** | **Summary of findings** | **References** |
| --- | --- | --- | --- | --- | --- |
| UTAUT and TTF | Automotive | South Korea | None | Both UTAUT-TTF can server an essential role in predicting consumer behaviour. | Kim et al. (2022) |
|  | Banking | Lebanon and China | Probability and Non-probability | The outcomes highlight that both UTAUT and TTF are essential theoretical framework to explain the mobile banking user adoption. | Tarhini et al. (2016) & Zhou et al. (2010) |
|  | Construction | China | None | The constructs of TTF have significant association with UTAUT variables. | Cai et al. (2023) and Hilal et al. (2019) |
|  | Education | India, China, Malaysia | Probability and Non-probability | The integration of information system theories (UTAUT-TTF) promote students active learning and enable them to exchange information and knowledge more efficiently. | Shirolkar and Kadam (2023), Tian and Yang (2023), Al-Rahmi et al. (2022), Al-Rahmi et al. (2022), Wang et al. (2022), Wan et al. (2020) and Chang (2013) |
|  | Healthcare | China, Turkey, Iran, South Korea, Pakistan | Probability and Non-probability | The findings indicated that UTAUT and TTF help to understand the user intention to adopt healthcare services. | Uymaz et al. (2023), Abdekhoda et al. (2022), Kang et al. (2022), Gu et al. (2021), Wang et al. (2020) |
|  | Retail | USA | Probability | Both frameworks (UTAUT and TTF) positively explain the consumers intention to adopt technology-driven products. | Park et al. (2015) |
|  | Public sector | Pakistan, Jordan, Yemen | Probability and Non-probability | Both UTAUT and TTF are the stronger predictors of BI than either UTAUT or TTF alone. | Alkhwaldi et al. (2022), Shahbaz et al. (2021) and Isaac et al. (2019) |
|  | Services | Tiawan | None | Both theoretical frameworks are important to adopt new and innovative technologies. | Pai and Tu (2011) |

The section of theoretical framework has completely revise with updated references from Pg-4-6, lines- 108-186 and for Table-1 on Pg- 20-21.

- Clarification on constructs (effort expectancy)

I understand your concern about the ambiguity surrounding the term "effort expectancy." To provide clarity, the term is specifically used in the context of adoption, reflecting users' perceived ease of use and the effort required to adopt the technology under investigation. This clarification has been added to the relevant sections of the manuscript to avoid any potential misunderstanding. Additionally, the following table explain the constructs in the theoretical model.

| **Constructs** | **Definition** | **Construct sources** | **Dimension involved** | **Factors related to the constructs** | **Reference** |
| --- | --- | --- | --- | --- | --- |
| Performance expectancy | PE refers to the degree to which individual believe that utilizing the technology will empower them to achieve optimal improvement in job performance. | UTAUT | Technology | Perceived usefulness, and view on performance | Shin et al., (2022), Abdelmageed & Zayad (2020), and Cai et al., (2023) |
| Effort expectancy | EE pertains to the ease of effort associated with the utilization of technology. | UTAUT | Technology | Perceived ease of use, technical complexity, and additional efforts | Cai et al., (2023), Jang et al., (2022) |
| Facilitating condition | FC involve the extent to which individuals are confident that the current organizational and technical infrastructure is in place to support the utilization of the technology. | UTAUT | Technology/organization | Experience and skills, R&D, and organizational structure | Zakaria et al., (2018), Luo et al., 2019) |
| Social influence | The belief that individual actions related to the use of technology will be accepted and embraced by affluent individuals or peers is referred to as social influence. | UTAUT | Environment | Government policy, stakeholder norm, and industry chain | Wang et al. (2021), Mao et al. (2018) |
| Task Characteristics | Task characteristics encompasses the challenges that potential adopters need to manage when employing technology in a specific context. | TTF | Technology/Environment | Cost, standardization, and coordination | Wu et al. (2019), and Masood et al. (2021) |
| Technology Characteristics | Technology characteristics encompasses the benefits offered by the technology. | TTF | Technology | Relative advantage, and motivation | Hu et al. (2019), and Cai et al. (2023) |
| Task-Technology fit | The extent to which the technology is perceived to complete the particular tasks. | TTF | Technology | Compatibility, and suitability | Gao et al. (2018) |
| Adoption intention | The intention of (potential) adopters for technology adoption. | UTAUT | ------- | Willingness | Cai et al. (2023), and Khashan et al. (2023) |

We believe that these revisions will strengthen the overall theoretical framework and improve the clarity of the connections between different theories and concepts. I appreciate your diligence in reviewing my work and thank you for guiding me towards enhancing the quality of the research.

**Reviewer#2, Comment # 1:** **In Introduction section, I suggest the authors clearly addressed that why he/she carried out this study? The gap should be addressed in introduction. However, the authors refer to the gap, but more information is needed.**

**Author response: Thanks for highlighting the issues and concerns.**

**Author action:** Thank you for your insightful feedback. We appreciate your suggestion regarding the need for a more explicit articulation of the study's rationale and the addressed gap in the introduction. In response, we have revised the introduction to provide a clearer and more detailed explanation of why this study was undertaken and how it fills the identified gap in the existing literature. We trust that these modifications enhance the overall coherence and purpose of the manuscript.

According to Miles (2017), the research work has multiple gaps like literature, population, theoretical, evidence, practical-knowledge and methodological gaps. Miles (2017) further argued that researchers should highlight three to four gaps. Thus, this research will fill the literature, theoretical, population and evidence gaps.

- **Literature and evidence gap:** “*Conclusively, the global rise of EV adoption reveals a notable gap, especially in Middle East region, due to geopolitical and pandemic disruptions, emphasizing the urgency for targeted interventions to facilitate a more inclusive and sustainable EV transition.”* The statement has been added on Pg-3, line 71-73.
- **Population gap:** “*In a nutshell, despite the pressing need for Saudi Arabia to reduce CO emissions, there exists a significant gap in the country transition to EV, exacerbated by a historical dependence on fossil fuels, limited use of renewable energy in road transportation, and the challenges of aligning consumer incentives with the complex dynamics of technological transformation and consumer acceptance.”* The statement has been added on Pg-3, line 88-92.
- **Theoretical gap:** “*Amidst evolving EV adoption literature, there is a growing recognition for diverse framework beyond traditional theories, necessitating deeper exploration of individual and technology dynamics.”* The statement has been added on Pg-4, line 105-107. Furthermore, in the theoretical framework section a conclusive statement has been added on Pg-6, lines-192-194, “*Table 1. Nevertheless, there is a dearth of empirical evidence supporting the application of the integrated framework in the EVs domain. This points to a theoretical gap in previous research, which can be addressed by incorporating it into the current study to fill this void.*”

**Reviewer#2, Comment # 2:** **In the literature section, there are many study which addressed this issue. I suggest the authors mentioned them in literature review section.**

**Author response: Thanks for highlighting the issues and concerns.**

**Author action:** We have addressed your suggestion by incorporating a comprehensive literature review section within the theoretical framework. To provide a more detailed overview, we have also included supplementary material that features a literature review table, the same has also been added at the end of this document which offering a thorough examination of studies addressing the pertinent issue. We believe these additions strengthen the scholarly foundation of the manuscript.

**Reviewer#2, Comment # 3:** **I suggest the manuscript could be improved significantly by more description of findings and cite to more document in each specific section.**

**Author response: Thanks for your concern.**

**Author action:** We have revisited the manuscript and made substantial improvements by providing more detailed descriptions of our findings in each specific section. Additionally, we have expanded our citations to include a more comprehensive range of relevant documents, thereby enhancing the overall depth and robustness of our work. For instance, on Pg-13 & 14, lines 423-429, 442-445 and 447-450.

**Reviewer#2, Comment # 4:** **The contribution of knowledge is not clear, very well. The author should identify the lessons that we can learn from his/her study.**

**Author response: Thanks for your suggestions.**

**Author action:** Thank you for your thoughtful feedback. We acknowledge the importance of clarifying the knowledge contribution of our study. We have revised the theoretical contribution section on Pg-15, line 462-478 to explicitly identify the lessons derived from our research.

- The study makes significant contributions by broadening the understanding of electric vehicles (EVs) through the integration of TTF and UTAUT frameworks. This synthesis offers a fresh perspective beyond a single theoretical framework, addressing a recognized call in literature. Through a methodical empirical approach, the study enhances the field of technological acceptance models, providing a validated model with robust associations.
- Furthermore, our research contributes to the literature by examining specific challenges and opportunities in the Middle East region, particularly in Saudi Arabia, related to EV adoption. The study provides nuanced insights and proposes targeted interventions to facilitate a more inclusive and sustainable transition to EVs in this unique and dynamic environment.
- Finally, empirical research makes a significant contribution by addressing a theoretical gap in the EV adoption literature. The application of an integrated framework of UTAUT and TTF fills an existing void in research, enriching and validating the theoretical landscape. These contributions provide valuable insights for future research and practical applications in the EV domain.

We believe these clarifications emphasize the lessons and knowledge gained from our study. Thank you for your time and valuable feedback.

**Reviewer#2, Comment # 5:** **The originally / novelty of the study is not clear very well.**

**Author response: Thanks for your concerns.**

**Author action:** Thank you for your feedback. We appreciate the opportunity to clarify the novelty and originality of our study. To address this concern, we have revised both the theoretical and practical implication sections of the manuscript on Pg-15 & 16, lines-462 to 494.

- In the theoretical section, we have explicitly highlighted the identified gaps stated in the introduction and theoretical framework. The integration of TTF and UTAUT frameworks to offer a fresh perspective addresses the existing call in the literature and contributes to the theoretical landscape. Our revised theoretical framework emphasizes the unique approach taken to fill the identified gaps.
- Furthermore, in the practical implications section, we have underscored how the study's findings provide targeted interventions to address specific challenges and opportunities in the Middle East region related to EV adoption, aligning with the identified gaps in the introduction and theoretical sections. This alignment emphasizes the practical relevance and contributions of our research.

We trust that these revisions clarify the novelty and originality of our study by explicitly linking the identified gaps with our theoretical and practical contributions. Thank you for your time and valuable feedback.

**Reviewer#3, Comment # 1:** **The introduction should provide a clearer explanation of the study's contributions**

**Author response: Thanks for your concerns.**

**Author action:** Thank you for your feedback. We have carefully revised the introduction to provide a clearer and more detailed explanation of the study's contributions. The updated introduction explicitly outlines the unique contributions of our research, emphasizing the identified gaps in the literature and how our study fills these gaps by integrating TTF and UTAUT frameworks. We believe these revisions enhance the overall clarity of the study's contributions. The contributions statement has been added on Pg-4, line-116 to 124.

**Reviewer#3, Comment # 2:** **While the literature review effectively discusses relevant theories, it's crucial to include a section detailing how the model was constructed based on these theories.**

**Author response: Thanks for your concerns.**

**Author action:**

**Reviewer#3, Comment # 3:** **In the discussion section, clarify the reasons for either accepting or rejecting the hypotheses within the context, and support these decisions with reference to prior research. The discussion section requires further development.**

**Author response: Thanks for your concerns.**

**Author action:** We have addressed your suggestion by incorporating a comprehensive literature review section within the theoretical framework. To provide a more detailed overview, we have also included supplementary material that features a literature review table, the same has also been added at the end of this document which offering a thorough examination of studies addressing the pertinent issue. We believe these additions strengthen the scholarly foundation of the manuscript.

**Reviewer#3, Comment # 4:** **Consider creating a dedicated section to address both the theoretical and practical implications.**

**Author response: Thanks for your concerns.**

**Author action:** Thank you for your insightful suggestion. We have carefully considered your feedback and have created a dedicated section in the manuscript to address both the theoretical and practical implications of our study on Pg-15 & 16, lines 462-494. This new section aims to provide a more structured and comprehensive overview of the contributions our research makes to both theoretical advancements and practical applications.

**Reviewer#3, Comment # 5:** **Ensure a comprehensive conclusion that addresses the study's core questions.**

**Author response: Thanks for your concerns.**

**Author action:** We have included the conclusion section that addresses the core questions on Pg-16, line 514-520.

**Reviewer#3, Comment # 6:** **For enhancing the UTAUT theory section, you can refer to the following sources:**

- Al-Mamary, Y. H. S. (2022). "Understanding the use of learning management systems by undergraduate university students using the UTAUT model: Credible evidence from Saudi Arabia." Published in the International Journal of Information Management Data Insights, 2(2), 100092.
- Y. H. S., Siddiqui, M. A., Abdalraheem, S. G., Jazim, F., Abdulrab, M., Rashed, R. Q., ... & Aliyu Alhaji, A. (2023). "Factors impacting Saudi students’ intention to adopt learning management systems using the TPB and UTAUT integrated model." Published in the Journal of Science and Technology Policy Management.

**Author response: Thanks for your concerns.**

**Author action:** The valuable research documents have been added in the revised manuscript on Pg-17, line 541-546.

| **Sr#** | **Authors** | **Evidence type** | **Purpose** | **Theory** | **Industry type** | **Sampling** | **Unit of analysis** | **Country** | **Findings** |
| --- | --- | --- | --- | --- | --- | --- | --- | --- | --- |
| 1 | Liu et al. (2023) | Empirical | To study the patient attitudes towards the usage and adoption of accessible electronic health records. | TTF, UTAUT, and Delone & McLean IS success model | Healthcare | Random | Patients | China | The results indicated that performance expectance, effort expectance, social influence and TTF are essential predictors of behavioural intention. |
| 2 | Shirolkar and Kadam (2023) | Empirical | To study the adoption and utilization of online examination portal in Indian universities. | UTAUT and TTF | Education | Convenience | Student | India | The outcomes indicated that UTAUT factors have direct effect on student adoption and TTF has a moderating impact on the relationships. |
| 3 | Sediyaningsih et al. 2023 | Empirical | To study the usage intention of metaverse technology in digital library services. | UTAUT, Unified information theory, and TTF | Higher education |  | Staff & students | Indonesia | The results suggested that TTF and UTAUT and ISS theories have well-explained the intention to use metaverse in higher education. |
| 4 | Uymaz et al. (2023) | Empirical | To study the BI to use AI doctor at the individual level at primary, secondary and teritary care levels. | UTAUT and TTF | Healthcare |  | Individual staff | Turkey | The outcome depicted that TTF has significant linkages with UTAUT variables and BI. |
| 5 | Dayour et al. (2023) | Qualitative (Interview) | To study the impact of ICT services on SME tourism and hospitality enterprises in Ghana | UTAUT2, TTF, Diffusion of innovation | Tourism & hospitality | Purposive | Hotel owner/manager | Ghana | The findings depicted that adoption of IT leads to the sustainability and survival of SMEs in tourism and hospitality industry. |
| 6 | Tian and Yang (2023) | Empirical | To examine the student interpreters BI to use interpreting technology in China. | UTAUT and TTF | Education | Purposive | Student | China | The result indicated that TTF and UTAUT (performance expectancy) have the most significant relationship. |
| 7 | Cai et al. (2023) | Empirical | To study the process of high prefabrication level technologies adoption form the enterprise perspective in China | UTAUT and TTF | Construction |  | Professional | China | The constructs of TTF have significant association with UTAUT variables. |
| 8 | Hajj et al. (2023) | Empirical | To study the role of TTF, sense of virtual community and CI to use the E-government in Lebanon. | TTF, UTAUT and Sense of virtual community | Government | SRS | Public/citizen | Lebonen | The study indicated that UTAUT has a mediating role between TTF and sense of virtual community toward the adoption of e-government in Lebonen. |
| 9 | Khashan et al. (2023) | Empirical | To study the retailing customers adoption of augmented reality apps in Egypt. | UTAUT2 and TTF | Retailing | Snowball | Customer | Egypt | The outcomes depicted that TTF and UTAUT2 positively impact the customer BI to adopt AR apps in Egypt. |
| 10 | Alkhwaldi et al. (2022) | Empirical | To analyze the adoption of HR information systems in Jordan public sector. | UTAUT and TTF | Public sector | Convenience | HR managers | Jordan | The findings highlight that UTAUT variables have a direct impact on user intention to adopt HRIS in the public sector and TTF has a mediating role between UTAUT and adoption intention. |
| 11 | Sun and Guo (2022) | Empirical | To study the adoption of digital museum usage behavior in China. | UTAUT2 and TTF | Tourism | None | Tourist | China | Both UTAUT-TTF can server an essential role in predicting consumer digital museum use behaviour. |
| 12 | Kim et al. (2022) | Empirical | To investigate the drivers of buy online & pick-up store (BOPS) usage intention of omnichannel consumers in the automotive retail sector. | UTAUT and TTF | Automotive | None | Customers | South Korea | Both UTAUT-TTF can server an essential role in predicting consumer behaviour. |
| 13 | Al-Rahmi et al. (2022) | Empirical | To evaluate the role of social media for teaching & learning in higher education. | UTAUT and TTF | Education | Stratified random | University student | Malaysia | The integration of information system theories (UTAUT-TTF) promote students active learning and enable them to exchange information and knowledge more efficiently. |
| 14 | Wang et al. (2022) | Empirical | To study the students intention to use tablet computers in China | UTAUT and TTF | Education | Convenience | Student | China | The factors like hedonic motivation and TTF are predictors among village students and habit and TTF are the most significant factors in urban areas students. |
| 15 | Abdekhoda et al. (2022) | Empirical | To determine the factors in adoption of e-learning in healthcare. | UTAUT and TTF | Healthcare | Random | University faculty | Iran | The integration of UTAUT and TTF explicitly explain the faculty intention to adopt e-learning. |
| 16 | Kang et al. (2022) | Empirical | To examine the behavioural intention to adopt smart home healthcare services in South Korea. | UTAUT and TTF | Healthcare | Random | Customers | South Korea | The findings indicated that UTAUT and TTF help to understand the user intention to adopt smart healthcare services. |
| 17 | Gu et al. (2021) | Empirical | To examine the adoption of e-health technology in Pakistan. | UTAUT and TTF | Healthcare | None | Patients | Pakistan | The results indicated that adoption of e-health technology is mostly influenced by the factors of UTAUT and TTF. |
| 18 | Sani et al. (2021) | Empirical | To study the factors affecting the adoption of big data analytics. | UTAUT, TTF and Initial trust model | Public sector | Random | Employees | Malaysia | The empirical findings indicated that both UTAUT and TTF are valid frameworks to adopt big data analytics in Malaysian government agencies. |
| 19 | Shahbaz et al. (2021) | Empirical | To examine the adoption of big data analytics-environmental air pollution management systems. | UTAUT and TTF | Public sector | Random | Employees | Pakistan | Both UTAUT and TTF are the stronger predictors of BI than either UTAUT or TTF alone. |
| 20 | Wan et al. (2020) | Empirical | To study the student intention of use massive open online courses. | UTAUT and TTF | Education | Random | Student | China | The results indicated that the factors of UTAUT model have direct impact on student open course learning, and TTF has indirect effect on continued intention. |
| 21 | Zhao and Bacao (2020) | Empirical | To determine the adoption factors related to food delivery apps. | UTAUT, TTF, and Expectancy confirmation model | Food | None | Consumer | China | The findings show that both frameworks (UTAUT-TTF) have direct and indirect positive impacts on user intention for food delivery mobile application. |
| 22 | Wang et al. (2020) | Empirical | To examine and understand the consumer acceptance of healthcare wearable devices. | UTAUT and TTF | Healthcare | Convenience | Consumer | China | The empirical results indicated that UTAUT factors (PE. EE, FC & SI) and TTF positively impacts BI and together accounted 68% of its variance. |
| 23 | Alazab et al. (2020) | Empirical | To investigate the adoption of blockchain in supply chain management. | UTAUT-TTF, Information system success (ISS) model | Manufacturing | None | SC Managers | Australia | The results indicated that IS theories (UTAUT-TTF-ISS) positively affect the supply chain managers to adopt blockchain. |
| 24 | Sahid et al. (2020) | Empirical | To examine the factors affecting the IT professional behavioural intentions in adopting big data analytics in Malaysian public sectors. | UTAUT-TTF and Initial trust model | Public sector | Random | Employees | Malaysia | Both UTAUT and TTF are the stronger predictors of BI than either UTAUT or TTF alone. |
| 25 | Hilal et al. (2019) | Conceptual | To develop a hybrid model for building information modelling adoption in facilities management. | UTAUT and TTF | Construction | None | None | None | The outcomes indicated that the acceptance and adoption of building information modelling adoption in facilities management is improved by UTAUT and TTF. |
| 26 | Isaac et al. (2019) | Empirical | To examine the adoption of internet usage among Yemen government organizations. | UTAUT and TTF | Public sector | None | Employees | Yemen | The integration of UTAUT and TTF explicitly explains the government intention to adopt the internet, and both frameworks explain 29% of variance. |
| 27 | Paulo et al. (2017) | Empirical | To examine the adoption of mobile augmented reality in tourism. | UTAUT2 and TTF | Services | None | Customers | Portugal | The empirical findings highlight that both frameworks explain 72% variance to adopt augmented reality and 45% user behaviour. |
| 28 | Tarhini et al. (2016) | Empirical | To analyze the factors that facilitate or hinder the usage of internet banking in Lebanon. | UTAUT and TTF | Banking | Convenience | Customers | Lebonan | The findings highlight that performance expectancy, social influence, perceived credibility and TTF are the essential predictors to explain customer behavioural intention to use Lebanon banking services. |
| 29 | Afshand and Sharif (2016) | Empirical | To examine the mobile banking adoption among university students in Pakistan. | UTAUT-TTF and Initial trust model | Education | Convenience | University student | Pakistan | The results indicated that TTF, UTAUT and initial trust model (ITM) are important framework to explain the mobile banking adoption. |
| 30 | Park et al. (2015) | Empirical | To examine the factors that affect the consumers intentions to use a revolutionary technology-driven product. | UTAUT and TTF | Online retail | Random | Customers | USA | Both frameworks (UTAUT and TTF) positively explain the consumers intention to adopt technology-driven products. |
| 31 | Oliveira et al. (2014) | Empirical | To exaime the adoption of mobile banking in Portugal. | UTAUT-TTF and Initial trust model | Banking | None | Customers | Portugal | The results indicated that TTF, UTAUT and initial trust model (ITM) are important framework to explain the mobile banking adoption. |
| 32 | Chang (2013) | Empirical | To explain the student behavioral intention of using library mobile applications. | UTAUT and TTF | Education | Convenience | University student | Taiwan | The findings indicated that UTAUT has a direct affect on student behaviour, and TTF has a moderating role on the relationships. |
| 33 | Pai and Tu (2011) | Empirical | To investigate the acceptance and usage of customer relationship management systems in Taiwan service industry. | UTAUT and TTF | Services | None | Employees | Taiwan | Both theoretical frameworks are important to adopt new and innovative technologies. |
| 34 | Zhou et al. (2010) | Empirical | To examine the mobile banking adoption among Chinese customers. | UTAUT and TTF | Banking | Random | Customers | China | The outcomes highlight that both UTAUT and TTF are essential theoretical framework to explain the mobile banking user adoption. |
